# Prevalence and Correlates of Firearm Screening and Safety Counseling in Pediatric Primary Care

**DOI:** 10.1007/s10900-025-01487-1

**Published:** 2025-06-01

**Authors:** Joseph B. Ladines-Lim, Anjali Vaishnav, Caroline Hayse, Elise Corden, Candice Gard, Grace Luger, Justin Litzner, Megan Olson, Jennifer Stojan, Margeaux Naughton, Michelle Degli Esposti, Jennifer Meddings

**Affiliations:** 1 Departments of Internal Medicine and Pediatrics, Michigan Medicine, University of Michigan, 3116 Taubman Center, SPC 5368, 1500 E. Medical Center Drive, 48109 Ann Arbor, MI USA; 2 University of Michigan Medical School, 1301 Catherine St, 48109 Ann Arbor, MI USA; 3 Department of Pediatrics, Michigan Medicine, University of Michigan, Medical Professionals Building, 1522 Simpson Road East, 48109 Ann Arbor, MI USA; 4 Michigan Medicine, University of Michigan, 1500 E Medical Center Drive, 48109 Ann Arbor, MI USA; 5 Institute for Firearm Injury Prevention, University of Michigan, 48109 Ann Arbor, MI USA; 6 Center for Clinical Management Research at the Veterans’ Affairs Ann Arbor Healthcare System, North Campus Research Complex, 2800 Plymouth Rd, Building 16, 48109 Ann Arbor, MI USA; 7 Present Address: Division of Infectious Diseases, Department of Medicine, Penn Medicine, University of Pennsylvania, 3400 Spruce St, 3 Silverstein, Ste E, Philadelphia, PA 19104 USA

**Keywords:** Firearm injuries, Preventive services, Health services research, Children, Firearm screening

## Abstract

Firearm-related injuries remain the leading cause of mortality in children in the United States. It is not well-characterized how often clinicians perform firearms access screening and safety counseling. We examined documentation of these services in a cross-sectional study of well child exams (WCEs) at a tertiary academic center in Southeast Michigan. Overall, we found that clinicians documented screening in 25,469 of 32,582 WCE encounters due to categorical Pediatrics clinicians doing so in 73.8% of encounters; Family Medicine and Internal Medicine-Pediatrics clinicians documented some form of screening in nearly all (> 99%) encounters. Clinicians documented counseling in 21.8% of encounters with Family Medicine clinicians lagging the other two specialties (8.2% versus 23.9% and 18.4% for Pediatrics and Internal Medicine-Pediatrics, respectively). Multinomial logistic regression for screening (conducted only for Pediatrics given the near universal screening by Family Medicine and Internal Medicine-Pediatrics) and counseling (conducted for all specialties) showed decreased likelihood of both screening and counseling for certain age and sociodemographic groups; however, while there was decreased likelihood for non-attending physician clinicians (i.e. advanced practice providers and resident/fellow physicians) and high inter-clinic variability (77.1%) for screening, the opposite was true for counseling with decreased likelihood for attending physicians and inter-clinic variability of 16.7%. Findings suggest that quality improvement efforts and clinician training are needed to eliminate gaps in risk-stratified screening and counseling regarding firearm safety.

**Supplementary Information:**

The online version contains supplementary material available at 10.1007/s10900-025-01487-1.

## Introduction

Firearms-related injuries are the leading cause of childhood death in the United States [1]. Current American Academy of Pediatrics (AAP) guidelines recommend routine firearm access screening and safety counseling during visits, particularly for those at risk for self-harm [2]. However, clinicians face barriers such as time constraints and insufficient training [3, 4].

At our large tertiary academic center in Southeast Michigan, we evaluated how frequently clinicians perform firearm access screening and safety counseling by analyzing documentation during well child exams (WCEs) and assessing associations with patient and clinician characteristics.

## Methods

We conducted a retrospective cross-sectional analysis of WCE documentation for patients ≤ 21 years old across 18 Michigan Medicine clinics (Pediatrics, Internal Medicine-Pediatrics, and Family Medicine) from September 1, 2021, to February 28, 2022. Data included patient sociodemographic and clinical characteristics (age, sex, race/ethnicity, active medical problems), clinician specialty and training level, and documentation of firearm access screening and safety counseling. We used diagnosis codes to identify active comorbidities and calculate the Pediatric Comorbidity Index (PCI) (see **Appendix**) [5]. We treated age as an ordinal variable; all other covariates were categorical. The University of Michigan Medical School Institutional Review Board approved this study (study no. HUM00220241) with data de-identified and destroyed post-study. We followed STROBE guidelines for cross-sectional studies.

Outcomes included documented firearm access screening and safety counseling. We used multinomial logistic regression to estimate associations between outcomes and patient/clinician characteristics, with multilevel modeling for patients nested within clinic sites and two-sided hypothesis tests (α = 0.05). We used RStudio version 4.2.2 (R Foundation for Statistical Computing, Vienna, Austria).

## Results

Overall, clinicians documented screening in 25,469 of 32,582 WCE encounters (Table 1). Screening prevalence was 73.8% in Pediatrics, 99.7% in Family Medicine, and 99.9% in Internal Medicine-Pediatrics. Clinicians documented counseling in 21.8% of encounters; Pediatrics, Family Medicine, and Internal Medicine-Pediatrics had prevalences of 23.9%, 8.2%, and 18.4%, respectively.

**Table 1:** Sample characteristics and primary and secondary outcomes

Characteristic	No. (%)			
	Overall (N = 32,582)	Pediatrics (N = 27,061)	Family Medicine (N = 3,711)	Internal Medicine-Pediatrics (N = 1,810)
Female sex	16,007 (49.1%)	13,299 (49.1%)	1,808 (48.7%)	900 (49.7%)
*Age group, years*				
0–1	9,627 (29.5%)	8,039 (29.7%)	1,083 (29.2%)	505 (27.9%)
2–5	11,107 (34.1%)	9,316 (34.4%)	1,231 (33.2%)	560 (30.9%)
6–11	6,694 (20.5%)	5,521 (20.4%)	795 (21.4%)	378 (20.9%)
12–21	5,154 (15.8%)	4,185 (15.5%)	602 (16.2%)	367 (20.3%)
*Race/ethnicity*				
Hispanic, any race	2,206 (6.8%)	1,714 (6.3%)	348 (9.4%)	144 (8.0%)
Non-Hispanic, Asian	2,572 (7.9%)	1,656 (6.1%)	706 (19.0%)	210 (11.6%)
Non-Hispanic, Black	3,652 (11.2%)	2,897 (10.7%)	545 (14.7%)	210 (11.6%)
Non-Hispanic, Other	3,019 (9.3%)	2,496 (9.2%)	314 (8.5%)	209 (11.5%)
Non-Hispanic, White	21,133 (64.9%)	18,298 (67.6%)	1,798 (48.5%)	1,037 (57.3%)
*History of psychiatric disorder, suicide attempt, * *suicidal or homicidal ideation, or substance use disorder*				
History of psychiatric disorder	2,977 (9.1%)	2,446 (9.0%)	307 (8.3%)	224 (12.4%)
History of suicide attempt or suicidal or homicidal ideation	54 (0.2%)	43 (0.2%)	8 (0.2%)	3 (0.2%)
History of substance use disorder	59 (0.2%)	53 (0.2%)	3 (0.1%)	3 (0.2%)
*Pediatric comorbidity index*				
0	21,467 (65.9%)	17,678 (65.3%)	2,615 (70.5%)	1,174 (64.9%)
≥ 1	11,115 (34.1%)	9,383 (34.7%)	1,096 (29.5%)	636 (35.1%)
*Level of clinician conducting the visit*				
Attending physician	26,306 (80.7%)	22,374 (82.7%)	2,621 (70.6%)	1,311 (72.4%)
Resident or fellow physician	5,760 (17.7%)	4,681 (17.3%)	596 (16.1%)	483 (26.7%)
Advanced practice provider	516 (1.6%)	6 (0.0%)	494 (13.3%)	16 (0.9%)
*Outcome*				
Screening	25,469 (78.2%)	19,959 (73.8%)	3,701 (99.7%)	1,809 (99.9%)
Positive screen	3,583 (14.1%)	3,225 (16.2%)	198 (5.3%)	160 (8.8%)
Negative screen	12,619 (49.5%)	11,017 (55.2%)	1,025 (27.7%)	577 (31.9%)
No response	9,267 (36.4%)	5,717 (28.6%)	2,478 (67.0%)	1,072 (59.3%)
Counseling	7,115 (21.8%)	6,478 (23.9%)	304 (8.2%)	333 (18.4%)
Positive screening	1,712 (24.1%)	1,634 (25.2%)	39 (12.8%)	39 (11.7%)
Negative screening	3,709 (52.1%)	3,513 (54.2%)	71 (23.4%)	125 (37.5%)
Lack of screening response	1,694 (23.8%)	1,331 (20.5%)	194(63.8%)	169 (50.8%)

Denominator of screening responses is total number of any screening, rather than the entire sample. Denominator of any counseling is entire sample. Denominator of counseling with any screening or counseling with no screening is total number of any counseling. Denominator of counseling with positive screening, negative screening, or lack of response to screening is total number of counseling with any screening

**Fig. 1:**
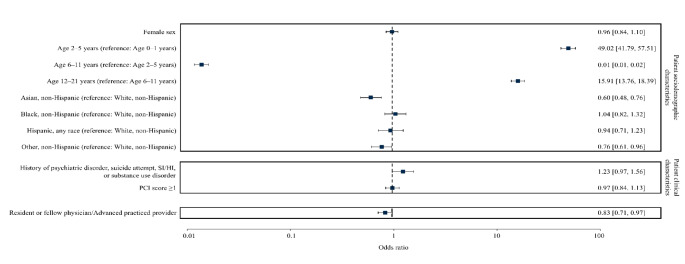
Odds ratios of logistic regression model correlates of firearm screening for Pediatrics clinicians. Abbreviations: PCI = Pediatric Comorbidity index; SI = suicidal ideation; HI = homicidal ideation. This was a Pediatrics-only sub-analysis given that Family Medicine and Internal Medicine-Pediatrics clinicians had almost all WCEs associated with screening. Adjusted odds ratios with 95% confidence intervals in brackets are shown on the right

We conducted multinomial regression analysis of screening for Pediatrics only given near-universal documented screening by Family Medicine and Internal Medicine-Pediatrics (Fig. 1). There was decreased screening likelihood for ages 0–1 and 6–11 years, patients identified as non-Hispanic Asian or “Other,” and care by resident/fellow physicians or advanced practice providers (APPs). Inter-clinic variability accounted for 77.1% of screening differences.

**Fig. 2:**
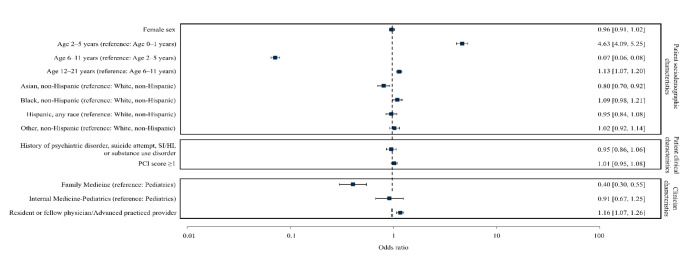
Odds ratios of logistic regression model correlates of firearm safety counseling for Pediatric, Family Medicine, and Internal Medicine-Pediatrics clinicians. Abbreviations: PCI = Pediatric Comorbidity index; SI = suicidal ideation; HI = homicidal ideation. Adjusted odds ratios with 95% confidence intervals in brackets are shown on the right

Regression analysis for counseling, including all specialties (Fig. 2), revealed decreased likelihood for ages 0–1 and 6–11 years, non-Hispanic Asian patients, Family Medicine clinicians, and attending physicians, with 16.7% of variability attributable to inter-clinic differences.

## Discussion

In this cross-sectional analysis of WCEs at a large tertiary medical center in Southeast Michigan, clinicians commonly documented firearm screening but not counseling. Contrary to AAP recommendations, clinician training level and patient sociodemographic characteristics, rather than high-risk clinical features such as psychiatric history, influenced outcomes [2, 6]. Marked differences in both screening and counseling frequency by age range suggest age-based questionnaire distribution and screening and counseling practices that pervade the health system.

Interestingly, Pediatrics clinicians screened less often than Family Medicine and Internal Medicine-Pediatrics clinicians while Family Medicine clinicians counseled least often. Although attending physicians appeared to screen more frequently, they also appeared to counsel less frequently compared with resident/fellow physicians and APPs. Possible reasons for these discrepancies include differences in practice across clinics and specialties within the institution, as well as variability across individual providers in relevant training, comfort level addressing the issue, and subsequent personal practice. The relatively low inter-clinic variability in counseling compared to that in screening suggests that while screening practices vary widely across pediatricians, clinicians of all specialty types in our study (Pediatrics, Family Medicine, and Internal Medicine-Pediatrics) may be more intentionally providing counseling when warranted, albeit at much lower rates.

Our findings add to the existing literature on screening and counseling practices regarding firearm injury prevention. One study found that 28% of families owned at least one firearm, with 11% endorsing having discussed the issue with their pediatrician, based on direct parent surveys [7]. A more recent quality improvement study found baseline rates of gun safety discussions during WCEs based on documentation to be 3% in a pediatric resident continuity clinic, with improvement to over 75% after implementation of an electronic health record prompt [8]. Still another study of resident clinics in Baltimore found very low screening and risk reduction counseling overall [9]. Other studies have focused on the adult population: for example, one study used natural language processing and machine learning approaches to identify charts with any mention of firearms in the veteran population and found a relatively low proportion of those with positive access to firearms (24.4%) [10], while another study found that 17.1% of survey respondents from five different states had been asked about firearm access by a healthcare provider [11]. While screening and counseling have been evaluated through different approaches, i.e., evidence of documentation [8–10], as our own study uses, versus survey-based responses [7, 11], our findings taken together with prior literature show great heterogeneity across study settings, not only in actual prevalence of screening and counseling but also in how these are evaluated in research.

Study limitations include data from a single institution and reliance on diagnosis codes to characterize medical history which may not be updated. Our results, found at a Midwestern academic medical center, may also not apply to other settings, such as private practices or other geographic regions. We also relied on documentation, which may not reflect actual practice; in particular, we note that counseling was often done even without a screening response, suggesting the possibility of documenting counseling from standardized templates without implementing this in practice. We also only studied WCEs, excluding other encounters that could address firearm screening and safety counseling, such as mental health encounters.

Overall, to ensure equitable firearm screening and safety counseling with appropriate risk stratification, findings highlight the need for quality improvement standardization efforts across clinics and specialties and individual clinician training [3, 9] which could incorporate existing, well-studied educational resources [2, 4, 12–14].

## Electronic Supplementary Material

Below is the link to the electronic supplementary material.

Supplementary Material 1

Supplementary Material 2

Supplementary Material 3

Supplementary Material 4

Supplementary Material 5

Supplementary Material 6

Supplementary Material 7

Supplementary Material 8

Supplementary Material 9

Supplementary Material 10

Supplementary Material 11

Supplementary Material 12

Supplementary Material 13

Supplementary Material 14

Supplementary Material 15

Supplementary Material 16

Supplementary Material 17

Supplementary Material 18

Supplementary Material 19

Supplementary Material 20

Supplementary Material 21

Supplementary Material 22

Supplementary Material 23

Supplementary Material 24

Supplementary Material 25

## Data Availability

Raw data was destroyed upon completion of the analysis in accordance with Institutional Review Board approval. Processed data that have had any personal identifiers removed is available upon request.
