# Supplementary material for: Prevalence and Correlates of Firearm Screening and Safety Counseling in Pediatric Primary Care
Source: J Community Health. 2025 Jun 1;50(5):959–64. doi: 10.1007/s10900-025-01487-1 (PMC12474701; doi:10.1007/s10900-025-01487-1)
Supplement: Supplementary file 25 — Supplementary Material 25 [file 10900_2025_1487_MOESM25_ESM.docx]

**Title:** Prevalence and Correlates of Firearm Screening and Safety Counseling in Pediatric Primary Care

**Journal:** Journal of Community Health

**Authors:**

Joseph B. Ladines-Lim, MD, PhD. Affiliation and address: Departments of Internal Medicine and Pediatrics, Michigan Medicine, University of Michigan, 3116 Taubman Center, SPC 5368, 1500 E. Medical Center Drive, Michigan, 48109. Email: joseph.ladines-lim@pennmedicine.upenn.edu

Anjali Vaishnav, BS. Affiliation and address: University of Michigan Medical School, 1301 Catherine St, Ann Arbor, Michigan, 48109. Email: anjaliv@med.umich.edu

Caroline Hayse, BS. Affiliation and address: University of Michigan Medical School, 1301 Catherine St, Ann Arbor, Michigan, 48109. Email: cahayse@med.umich.edu

Elise Corden, MD, MBA. Affiliation and address: Department of Pediatrics, Michigan Medicine, University of Michigan, Medical Professionals Building, 1522 Simpson Road East, Ann Arbor, Michigan, 48109. Email: ecorden@med.umich.edu

Candice Gard, MD. Affiliation and address: Department of Pediatrics, Michigan Medicine, University of Michigan, Medical Professionals Building, 1522 Simpson Road East, Ann Arbor, Michigan, 48109. Email: candicga@med.umich.edu

Grace Luger, MD. Affiliation and address: Department of Pediatrics, Michigan Medicine, University of Michigan, Medical Professionals Building, 1522 Simpson Road East, Ann Arbor, Michigan, 48109. Email: lugerg@med.umich.edu

Justin Litzner, BS. Affiliation and address: Michigan Medicine, University of Michigan, 1500 E Medical Center Drive, Ann Arbor, Michigan, 48109. Email: jlitzner@umich.edu

Megan Olson, MD. Affiliation and address: Department of Pediatrics, Michigan Medicine, University of Michigan, Medical Professionals Building, 1522 Simpson Road East, Ann Arbor, Michigan, 48109. Email: maolson@med.umich.edu

Jennifer Stojan, MD, MHPE. Affiliation and address: Departments of Internal Medicine and Pediatrics, Michigan Medicine, University of Michigan, 3116 Taubman Center, SPC 5368, 1500 E. Medical Center Drive, Michigan, 48109. Email: jstojan@med.umich.edu

Margeaux Naughton, MD. Affiliation and address: Department of Pediatrics, Michigan Medicine, University of Michigan, Medical Professionals Building, 1522 Simpson Road East, Ann Arbor, Michigan, 48109. Email: mreizian@med.umich.edu

Michelle Degli Esposti, PhD. Affiliation and address: Institute for Firearm Injury Prevention, University of Michigan, Ann Arbor, Michigan, 48109. Email: dr.m.degliesposti@gmail.com

Jennifer Meddings, MD, MSc. Affiliation and address: Center for Clinical Management Research at the Veterans’ Affairs Ann Arbor Healthcare System, North Campus Research Complex, 2800 Plymouth Rd, Building 16, Ann Arbor, Michigan, 48109. Email: meddings@med.umich.edu

**Corresponding author**: Joseph B. Ladines-Lim, MD, PhD. Present address: 3400 Spruce St, 3 Silverstein, Ste E, Philadelphia, Pennsylvania 19104. E-mail: joseph.ladines-lim@pennmedicine.upenn.edu. Phone: 267-581-2092. Fax: 215-662-7611.

**SUPPLEMENT**

We extracted data from our electronic medical record using the Epic Clarity database. We describe the variables used in our analysis below.

Patient sociodemographic and clinical characteristics:

1. Age in years
2. Biologic sex: female or male.
3. Race/ethnicity: Hispanic, any race; non-Hispanic Asian; non-Hispanic Black; non-Hispanic Other; non-Hispanic White.
4. Active medical problems based on those listed in problem list at time of encounter. We used ICD-10 diagnosis codes to map each problem to one of the diagnosis categories in the Pediatric Comorbidity Index (PCI). These codes are listed in the following Excel files which are also attached separately:
   1. ICD-10-CM_peds_alcohol_abuse_dependence.xlsx
   2. ICD-10-CM_peds_anemia.xlsx
   3. ICD-10-CM_peds_anxiety_panic_disorder.xlsx
   4. ICD-10-CM_peds_any_malignancy.xlsx
   5. ICD-10-CM_peds_asthma.xlsx
   6. ICD-10-CM_peds_cardiovascular_conditions.xlsx
   7. ICD-10-CM_peds_chromosomal_anomalies.xlsx
   8. ICD-10-CM_peds_conduct_disorder.xlsx
   9. ICD-10-CM_peds_congenital_malformations.xlsx
   10. ICD-10-CM_peds_depression.xlsx
   11. ICD-10-CM_peds_developmental_delays.xlsx
   12. ICD-10-CM_peds_diabetes_mellitus.xlsx
   13. ICD-10-CM_peds_drug_abuse_dependence.xlsx
   14. ICD-10-CM_peds_eating_disorders.xlsx
   15. ICD-10-CM_peds_epilepsy_convulsions.xlsx
   16. ICD-10-CM_peds_GI_conditions.xlsx
   17. ICD-10-CM_peds_joint_disorders.xlsx
   18. ICD-10-CM_peds_menstrual_disorders.xlsx
   19. ICD-10-CM_peds_nausea_vomiting.xlsx
   20. ICD-10-CM_peds_pain_conditions.xlsx
   21. ICD-10-CM_peds_psychotic_disorders.xlsx
   22. ICD-10-CM_peds_sleep_disorders.xlsx
   23. ICD-10-CM_peds_smoking.xlsx
   24. ICD-10-CM_peds_weight_loss.xlsx

Of note, we also mapped codes to the following categories: psychiatric disorder, substance use, and history of self-harm or suicidal ideation or homicidal ideation. These shared some but not complete overlap with some of the categories used for the PCI. The Excel files used for this purpose are listed below and also attached separately:

1. ICD-10-CM_psychiatric.xlsx
2. ICD-10-CM_substance_use.xlsx
3. ICD-10-CM_SHSIHI.xlsx

Clinician characteristics:

1. Training level: attending physician, resident or fellow physician, and advanced practice provider.
2. Specialty: Categorical Pediatrics, Family Medicine, or combined Internal Medicine-Pediatrics.

Of note, we used EMERSE to search through documentation to identify outcomes. We used this as an adjunctive method, not the sole method for identifying outcomes. We used the following terms for WCEs for patients included in the cohort: firearm*, gun*, weapon*.
